# Increased fat mass negatively influences femoral neck bone mineral density in men but not women

**DOI:** 10.3389/fendo.2023.1035588

**Published:** 2023-02-28

**Authors:** Nipith Charoenngam, Caroline M. Apovian, Chatlert Pongchaiyakul

**Affiliations:** ^1^ Department of Medicine, Mount Auburn Hospital, Harvard Medical School, Cambridge, MA, United States; ^2^ Department of Medicine, Faculty of Medicine Siriraj Hospital, Mahidol University, Bangkok, Thailand; ^3^ Department of Medicine, Division of Endocrinology, Diabetes, and Hypertension, Brigham and Women’s Hospital and Harvard Medical School, Boston, MA, United States; ^4^ Division of Endocrinology and Metabolism, Department of Medicine, Faculty of Medicine, Khon Kaen University, Khon Kaen, Thailand

**Keywords:** bone mineral density, fat mass, lean mass, osteoporosis, BMD

## Abstract

**Background:**

Obesity is known to be a protective factor against osteoporosis. However, recent studies have shown that excessive adiposity may be detrimental for bone health.

**Objective:**

To determine the association of lean mass (LM) and fat mass (FM) with bone mineral density (BMD) in Thais.

**Methods:**

Bone density studies of consecutive patients of Srinagarind Hospital, Khon Kaen, Thailand between 2010 and 2015 were reviewed. LM, FM, lumbar spine (LS) and femoral neck (FN) BMD were measured. Lean mass index (LMI) and fat mass index (FMI) were calculated [LMI=LM (kg)/height (m)^2^, FMI=FM (kg)/height (m)^2^] and analyzed to determine the association with LS and FN BMD using multiple regression analysis. This study was approved by the institutional ethical committee (HE42116).

**Results:**

A total of 831 participants were included. The mean ± SD age was 50.0 ± 16.3 years. In men, LMI (per 1 kg/m^2^ increase) was positively correlated with FN BMD (g/cm^2^, β 0.033) and LS BMD (g/cm^2^, β 0.031), after adjusting for age, height and FMI. Whereas FMI (per 1 kg/m^2^ increase) was negatively correlated with FN BMD (g/cm^2^, β -0.015) but not with LS BMD (g/cm^2^, β 0.005) after adjusting for age, height and LMI. In women, both LMI and FMI were positively correlated with LS BMD (g/cm^2^, LMI: β 0.012; FMI: β 0.016) and FN BMD (g/cm^2^, LMI: β 0.034; FMI: β 0.007) with age, height, LMI and FMI included in the model.

**Conclusion:**

Our findings indicate that FM has a sex-specific influence on BMD in Thais.

## Introduction

Osteoporosis, a condition characterized by bone fragility secondary to low bone mass and loss of connectivity and structural integrity of bone tissue, is the most common metabolic bone disease that affects over 200 million people worldwide (1, 2). It is estimated that one in every three women over the age of 50 years and one in every five men will suffer from fragility fractures as a result of osteoporosis during their lifetime (3). Traditional risk factors for osteoporosis include advanced age, female sex, family history, low calcium intake, malabsorption, vitamin D deficiency, lack of physical activity, weight loss, smoking, excessive alcohol use, and the presence of chronic inflammatory diseases (4). On the other hand, increased body weight and obesity have long been thought to be a protective factor against osteoporosis (4, 5).

Interestingly, recent evidence suggests that excess fat mass (FM) may be detrimental for bone health, as recent studies have found an inverse relationship between FM and bone mineral density (BMD), whereas previous studies found the opposite (6–9). Given the inconsistencies of the data, it is assumed that the relationship between FM and BMD is complex and different across sex and sites of BMD measurements (5, 6, 10). Therefore, we aimed to investigate the association of lean mass (LM) and fat mass (FM) with lumbar spine (LS) and femoral neck (FN) BMD in Thai men and women.

## Methods

### Study population

Bone density studies of male and female consecutive community-dwelling patients aged 20 – 90 years were retrospectively reviewed from the medical record database of Srinagarind Hospital, Khon Kaen, Thailand between 2010 and 2015. Participants aged 20 to 90 years who underwent BMD testing at both the lumbar spine and the hip were included in this study. Patients with one of the following exclusion criteria were excluded: history of fragility fractures at any sites; history of traumatic fractures of the spine or femur; medications that may affect bone metabolism except calcium and vitamin D; history of any spinal surgery; lumbar scoliosis greater than 20 degrees; two or more non-assessable lumbar vertebrae; early or surgical menopause; and Z-score outside the range of ± 2.0 at either the lumbar spine, total proximal femur, or the femoral neck. This study was reviewed and approved by the Khon Kaen University Human Research Ethics Committee in accordance with the Helsinki Declaration and the Good Clinical Practice Guidelines (Reference No. HE42116).

### Study measurements

Demographic data were collected including age, body weight, height, and body mass index (BMI) was calculated. Lumbar spine (LS), femoral neck (FN) BMD, lean mass (LM) and fat mass (FM) were measured using dual energy x-ray absorptiometry on a Lunar Prodigy bone densitometer (GE Healthcare, Madison, WI). Lean mass index (LMI) and fat mass index (FMI) were calculated [LMI=LM (kg)/height (m)^2^, FMI=FM (kg)/height (m)^2^] and were analysed to determine the association with LS and FN BMD using multiple regression analysis.

### Statistical analysis

Comparisons of participants’ characteristics between males and females were performed using independent sample t-test for continuous parametric data, Mann Whitney U-test for continuous non-parametric data and Chi-square test for categorical data. Comparisons of participants’ characteristics among groups with different LMI and FMI were performed using one-way ANOVA followed by post-hoc LSD and Bonferroni tests for continuous parametric data. Pearson correlation analysis was used to determine univariate association of age with LM, FM, LMI, FMI and FN and LS BMD. Linear regression analysis was performed to determine univariate and multivariate association of LMI and FMI with FN and LS BMD. Logistic regression analysis was used to determine unadjusted and adjusted odds ratios (OR) and 95% confidence interval (CI) that represent the association of LMI and FMI with osteoporosis at FN and LS. Statistical significance was defined as p-value <0.05. SPSS version 27 (SPSS Inc., Chicago, IL) was used to perform statistical analysis. Data illustrations were generated using the GraphPad Prism software 9.4.0 (GraphPad, La Jolla, CA, USA).

## Results

### Characteristics of participants

A total of 831 participants were included in the study. As demonstrated in **Table 1**, the mean ± SD age was 50.0 ± 16.3 years and 498 (59.9%) were female. The mean ± SD FN and LS BMD were 0.866 ± 0.177 g/cm^2^ and 1.060 ± 0.191 g/cm^2^, respectively. There were 333 (40.1%) and 55 (6.6%) participants with osteopenia (T-score FN BMD -1 to -2.5) and osteoporosis (T-score FN BMD ≤-2.5) of the FN, respectively. There were 229 (27.6%) and 120 (4.4%) participants with osteopenia and osteoporosis of the LS, respectively. The mean ± SD BMI, FM, LM, FMI and LMI were 23.3 ± 3.7 kg/m^2^, 15.5 ± 7.7 kg, 38.9 ± 8.0 kg, 6.4 ± 3.3 kg/m^2^, 15.6 ± 2.3 kg/m^2^, respectively. As shown in **Table 1**, female participants had statistically significantly lower BMI, FN and LS BMD, LM and LMI; higher FM and FMI; and higher proportion of osteoporosis compared with male participants (all p <0.001). Age was positively correlated with FM (R = 0.143, p <0.001) and FMI (R = 0.168, p <0.001) and was negatively correlated with LM (R = -0.191, p <0.001), LMI (R = -0.107, p = 0.002), FN BMD (R = -0.576, p <0.001) and LS BMD (R = -0.421, p <0.001).

**Table 1:** Characteristics of participants stratified by sex.

	All participants	Males	Females	p-value
	N = 831	N = 333 (40.1%)	498 (59.9%)	
Age (years)	50.0 ± 16.3	49.3 ± 17.3	50.5 ± 15.5	0.337
Body Weight (kg)	57.8 ± 10.3	60.9 ± 10.3	55.7 ± 9.8	<0.001
Height (cm)	157.3 ± 7.5	163.1 ± 6.4	153.4 ± 5.4	<0.001
Body mass index (kg/m^2^)	23.3 ± 3.7	22.8 ± 3.3	23.6 ± 3.9	<0.001
Body mass index <23 kg/m^2^	420 (50.5%)	192 (57.7%)	228 (45.8%)	0.001
Body mass index 23 - <25 kg/m^2^	183 (22.0%)	70 (21.0%)	113 (22.7%)	
Body mass index ≥25 kg/m^2^	228 (27.4%)	71 (21.3%)	157 (31.5%)	
FN BMD (g/cm^2^)	0.866 ± 0.177	0.920 ± 0.176	0.829 ± 0.168	<0.001
FN T-score	-0.8 ± 1.2	-0.8 ± 1.1	-0.9 ± 1.2	0.170
FN normal BMD (T-score >-1)	443 (53.3%)	181 (54.4%)	262 (52.6%)	0.134
FN osteopenia (T-score -1 – -2.5)	333 (40.1%)	137 (41.4%)	196 (39.4%)	
FN osteoporosis (T-score <-2.5)	55 (6.6%)	15 (4.5%)	40 (8.0%)	
LS BMD (g/cm^2^)	1.060 ± 0.191	1.111 ± 0.167	1.026 ± 0.200	<0.001
LS T-score	-0.8 ± 1.7	-0.4 ± 1.3	-1.1 ± 1.8	<0.001
LS normal BMD (T-score >-1)	482 (58.0%)	236 (70.9%)	246 (49.4%)	<0.001
LS osteopenia (T-score -1 – -2.5)	229 (27.6%)	81 (24.3%)	148 (29.7%)	
LS osteoporosis (T-score <-2.5)	120 (14.4%)	16 (4.8%)	104 (20.9%)	
Osteoporosis LS or FN (LS or FN T-score <-2.5)	132 (15.9%)	22 (6.6%)	110 (22.1%)	<0.001
Fat mass (kg)	15.5 ± 7.7	10.8 ± 6.1	18.6 ± 7.0	<0.001
Fat mass index (kg/m^2^)	6.4 ± 3.3	4.0 ± 2.2	7.9 ± 3.0	<0.001
% Body fat	27.9 ± 11.4	18.0 ± 7.9	34.5 ± 8.1	<0.001
Lean mass (kg)	38.9 ± 8.0	46.6 ± 6.0	33.8 ± 4.1	<0.001
Lean mass index (kg/m^2^)	15.6 ± 2.3	17.5 ± 1.8	14.4 ± 1.6	<0.001
% Lean mass	72.1 ± 11.4	82.0 ± 7.9	65.5 ± 8.1	<0.001

BMD, Bone mineral density; FN, Femoral neck; LS, Lumbar spine.

P-values were obtained from statistical testing of the difference between male and female participants.

### Femoral neck and lumbar spine T-score stratified by quartiles of lean mass index and fat mass index

**Figure 1:**
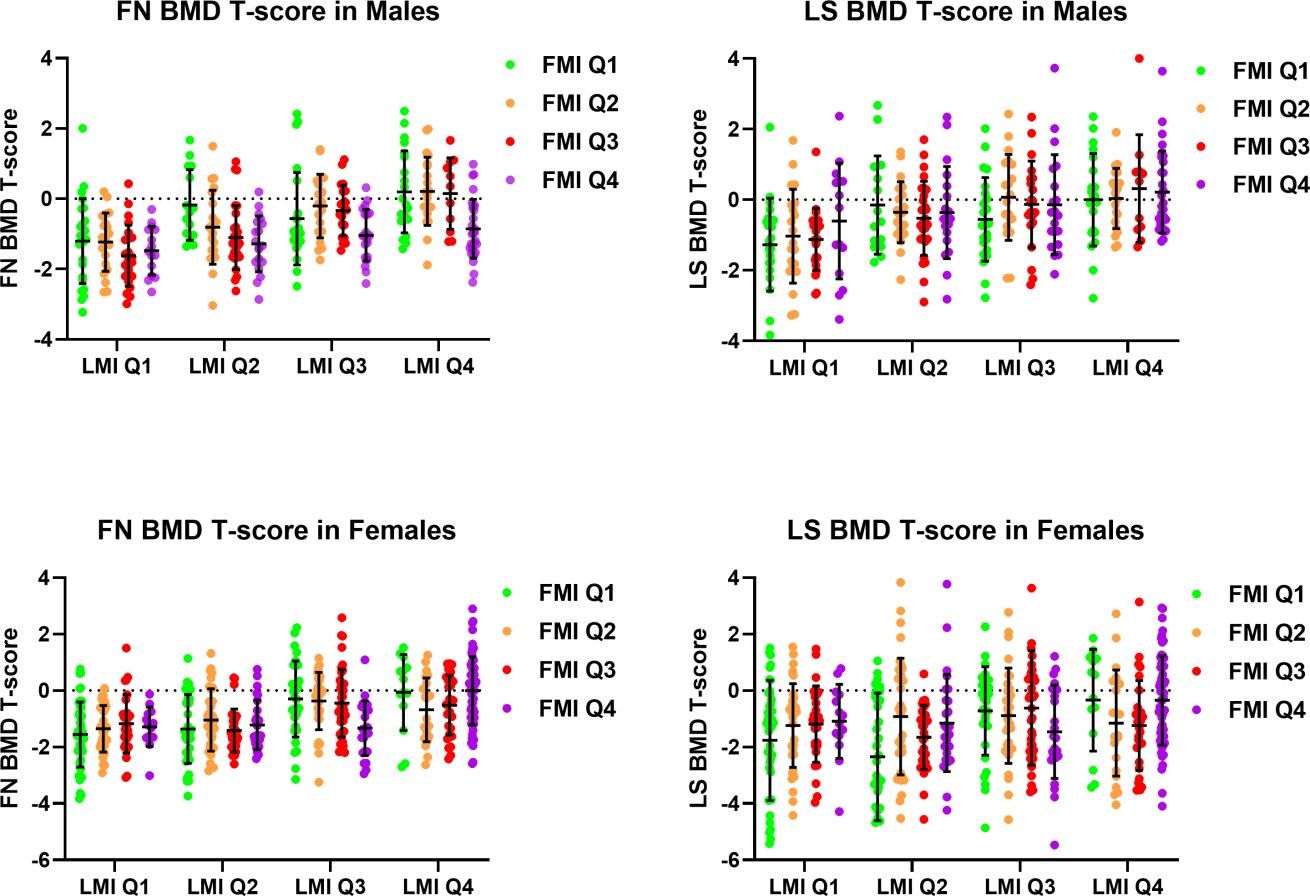
Femoral neck and lumbar spine bone mineral density T-score stratified by quartiles of lean mass index and fat mass index in male and female participants. BMD, Bone Mineral Density; FMI, Fat Mass Index; FN, Femoral Neck; LMI, Lean Mass Index; LS, Lumbar Spine.

**Figure 1** demonstrated mean FN and LS BMD T-score stratified by quartiles of LMI and FMI in male and female participants. The analysis of variance revealed significant differences in FN and LS T-score across the groups with different LMI among both male and female participants (all ANOVA p <0.01). FN T-scores were different across the groups with different FMI in male participants (ANOVA p <0.001), with post-hoc analysis revealing the Q1 FMI group having lower FN T-score than the Q4 FMI group (Bonferroni p <0.001), while the difference was not observed among the female participants (ANOVA p = 0.834). On the other hand, LS T-scores were not significantly different across the groups with different FMI in both male and female participants.

Subgroup analysis among women in the Q1 and Q2 LMI groups revealed a significant difference in LS T-score, but not FN T-score, across the groups with different FMI (ANOVA p <0.01 for LS T-score, ANOVA p = 0.384 for FN T-score), with post-hoc analysis showing a trend of lower LS T-score in the Q1 FMI group compared with the Q4 FMI group (LSD p = 0.012, Bonferroni p = 0.070). In the subgroup of women in the Q3 and Q4 LMI groups, no difference in LS or FN T-score across the groups with different FMI was observed.

### Association of lean mass index and fat mass index with femoral neck and lumbar spine bone mineral density

Regression coefficients of LMI and FMI on FN and LS BMD were demonstrated in **Table 2**. In male participants, FN BMD (g/cm^2^) was positively correlated with LMI (per 1 kg/m^2^ increase; β 0.033, 95%CI 0.024 – 0.041) and inversely correlated with FMI (per 1 kg/m^2^ increase; β -0.015, 95%CI -0.022 – -0.007), after adjusting for age and height with LMI and FMI included in the same model (Model 3, **Table 2**). Whereas LS BMD was associated with only increased LMI (per 1 kg/m^2^ increase; β 0.031, 95%CI 0.021 – 0.040) but not FMI (per 1 kg/m^2^ increase; β 0.005, 95%CI -0.003 – 0.014), with adjustment for the same variables (Model 3, **Table 2**).

**Table 2:** Regression coefficients of lean mass index and fat mass index on femoral neck and lumbar spine BMD.

	Unadjusted			Model 1			Model 2			Model 3		
	β	95%CI	p-value	β	95%CI	p-value	β	95%CI	p-value	β	95%CI	p-value
Femoral neck BMD (g/cm^2^)												
All participants												
LMI (per 1 kg/m^2^ increase)	0.034	0.029 – 0.039	<0.001	0.032	0.027 – 0.037	<0.001	0.029	0.022 – 0.037	<0.001	0.032	0.026 – 0.037	<0.001
FMI (per 1 kg/m^2^ increase)	-0.007	-0.010 – -0.03	<0.001	0.009	0.005 – 0.012	<0.001	-0.022	-0.029 – -0.015	<0.001	0.002	-0.001 – 0.006	0.221
Male participants												
LMI (per 1 kg/m^2^ increase)	0.036	0.027 – 0.045	<0.001	0.027	0.019 – 0.036	<0.001	0.046	0.033 – 0.059	<0.001	0.033	0.024 – 0.041	<0.001
FMI (per 1 kg/m^2^ increase)	-0.017	-0.026 – -0.009	<0.001	-0.006	-0.014 – -0.001	0.108	-0.048	-0.061 – -0.035	<0.001	-0.015	-0.022 - -0.007	<0.001
Female participants												
LMI (per 1 kg/m^2^ increase)	0.041	0.032 – 0.050	<0.001	0.038	0.031 – 0.045	<0.001	0.028	0.018 – 0.037	<0.001	0.034	0.027 – 0.041	<0.001
FMI (per 1 kg/m^2^ increase)	0.009	0.004 – 0.014	<0.001	0.014	0.010 – 0.018	<0.001	-0.014	-0.022 - -0.006	<0.001	0.007	0.003 – 0.010	<0.001
Lumbar spine BMD (g/cm^2^)												
All participants												
LMI (per 1 kg/m^2^ increase)	0.027	0.022 – 0.033	<0.001	0.023	0.016 – 0.029	<0.001	0.004	-0.005 – 0.013	0.386	0.018	0.011 – 0.025	<0.001
FMI (per 1 kg/m^2^ increase)	0.001	-0.003 – 0.005	0.640	0.018	0.014 – 0.022	<0.001	-0.003	-0.011 – 0.006	0.495	0.014	0.009 – 0.018	<0.001
Male participants												
LMI (per 1 kg/m^2^ increase)	0.032	0.023 – 0.041	<0.001	0.030	0.021 – 0.040	<0.001	0.024	0.010 – 0.038	0.001	0.031	0.021 – 0.040	<0.001
FMI (per 1 kg/m^2^ increase)	0.011	0.003 – 0.020	0.005	0.016	0.007 – 0.024	<0.001	-0.027	-0.042 – -0.013	<0.001	0.005	-0.003 – 0.014	0.209
Female participants												
LMI (per 1 kg/m^2^ increase)	0.024	0.013 – 0.035	<0.001	0.020	0.012 – 0.029	<0.001	-0.002	-0.014 – 0.009	0.699	0.012	0.003 – 0.021	0.010
FMI (per 1 kg/m^2^ increase)	0.012	0.007 – 0.018	<0.001	0.019	0.014 – 0.023	<0.001	0.006	-0.004 – 0.015	0.263	0.016	0.011 – 0.021	0.001

Model 1: adjustment for age and sex (for analysis of all participants); adjustment for age (for analysis of male and female participants).

Model 2: adjustment for age, sex, height and BMI (for analysis of all participants); adjustment for age, height and BMI (for analysis of male and female participants).

Model 3: adjustment for age, sex and height with LMI and FMI included in the same model (for analysis of all participants); adjustment with age and height, with LMI and FMI included in the same model (for analysis of male and female participants). BMI was removed from the model due to multicollinearity.

BMD, Bone mineral density; BMI, Body mass index; FMI, Fat mass index; LMI, Lean mass index.

In female participants, both LS and FN BMD (g/cm^2^) were positively correlated with LMI (per 1 kg/m^2^ increase; LS BMD: β 0.012, 95%CI 0.003 – 0.021; FN BMD: β 0.034, 95%CI 0.027 – 0.041) and FMI (per 1 kg/m^2^ increase; LS BMD: β 0.016, 95%CI: 0.011 – 0.021; FN BMD: β 0.007, 95%CI: 0.003 – 0.010), after adjusting for age, height and LMI and FMI included in the same model (Model 3, **Table 2**).

### Association of lean mass index and fat mass index with osteoporosis at femoral neck and lumbar spine

Multivariate logistic regression analysis of the association of LMI and FMI with presence of osteoporosis at FN and LS (defined by T-score BMD ≤-2.5) were demonstrated in **Table 3**. In male participants, LMI (per 1 kg/m^2^ increase) was statistically significantly associated with decreased odds of FN osteoporosis (OR 0.466, 95%CI 0.305 – 0.711) but not LS osteoporosis, after adjusting for age, height and FMI (Model 3, **Table 3**). FMI (per 1 kg/m^2^ increase) was statistically significantly associated with increased odds of FN osteoporosis (OR 2.037, 95%CI 1.132 – 3.666) after adjusting for age, height and BMI (Model 2, **Table 3**), but the association became insignificant in the model adjusting for age, height and LMI (Model 3, **Table 3**).

**Table 3:** Association of lean mass index and fat mass index with osteoporosis at femoral neck and lumbar spine.

	Unadjusted		Model 1		Model 2		Model 3	
	OR	95%CI	OR	95%CI	OR	95%CI	β	95%CI
All participants								
FN osteoporosis								
LMI (per 1 kg/m^2^ increase)	0.650	0.557 – 0.759	0.616	0.492 – 0.770	0.739	0.557 – 0.980	0.627	0.493 – 0.796
FMI (per 1 kg/m^2^ increase)	0.902	0.823 – 0.988	0.787	0.696 – 0.891	1.330	1.019 – 1.736	0.844	0.744 – 0.958
LS osteoporosis								
LMI (per 1 kg/m^2^ increase)	0.695	0.625 – 0.772	0.842	0.722 – 0.981	1.234	0.991 – 1.536	0.926	0.776 – 1.105
FMI (per 1 kg/m^2^ increase)	0.982	0.962 – 1.042	0.733	0.662 – 0.812	0.834	0.666 – 1.044	0.741	0.666 – 0.825
Male participants								
FN osteoporosis								
LMI (per 1 kg/m^2^ increase)	0.410	0.279 – 0.603	0.465	0.311 – 0.695	0.504	0.287 – 0.884	0.466	0.305 – 0.711
FMI (per 1 kg/m^2^ increase)	0.886	0.666 – 1.125	0.775	0.587 – 1.023	2.037	1.132 – 3.666	0.926	0.675 – 1.271
LS osteoporosis								
LMI (per 1 kg/m^2^ increase)	0.541	0.393 – 0.743	0.619	0.444 – 0.862	0.686	0.415 – 1.133	0.589	0.647 – 1.180
FMI (per 1 kg/m^2^ increase)	0.849	0.656 – 1.100	0.762	0.580 – 1.001	1.569	0.924 – 2.661	0.874	0.404 – 0.860
Female participants								
FN osteoporosis								
LMI (per 1 kg/m^2^ increase)	0.660	0.525 – 0.830	0.702	0.531 – 0.928	0.851	0.598 – 1.210	0.716	0.528 – 0.972
FMI (per 1 kg/m^2^ increase)	0.766	0.670 – 0.876	0.798	0.694 – 0.917	1.171	0.828 – 1.656	0.837	0.727 – 0.964
LS osteoporosis								
LMI (per 1 kg/m^2^ increase)	0.846	0.734 – 0.977	0.912	0.763 – 1.089	1.426	1.104 – 1.843	1.052	0.850 – 1.302
FMI (per 1 kg/m^2^ increase)	0.807	0.740 – 0.880	0.723	0.645 – 0.809	0.714	0.553 – 0.922	0.715	0.635 – 0.805

Model 1: adjustment for age and sex (for analysis of all participants); adjustment for age (for analysis of male and female participants). Model 2: adjustment for age, sex, height and BMI (for analysis of all participants); adjustment for age, height and BMI (for analysis of male and female participants). Model 3: adjustment for age, sex and height with LMI and FMI included in the same model (for analysis of all participants); adjustment with age and height, with LMI and FMI included in the same model (for analysis of male and female participants). BMI was removed from the model due to multicollinearity. FN and LS osteoporosis were defined as T-score of <-2.5 at each respective site. OR represents odds ratio of osteoporosis at femoral neck and lumbar spine per 1 kg/m2 increase in lean mass index and fat mass index.BMD, Bone mineral density; BMI, Body mass index; FMI, Fat mass index; FN, Femoral neck; LMI, Lean mass index; LS, Lumbar spine; OR, Odds ratio.

In female participants, LMI (per 1 kg/m^2^ increase) was statistically significantly associated with decreased odds of osteoporosis at FN (OR 0.716, 95%CI 0.528 – 0.972) but not LS, after adjusting for age, height and FMI. Whereas FMI (per 1 kg/m^2^ increase) was statistically significantly associated with decreased odds of osteoporosis at both FN (OR 0.837, 95%CI 0.727 – 0.964) and LS (OR 0.715, 95%CI 0.635 – 0.805), after adjusting for age, height and LMI (Model 3, **Table 3**).

## Discussion

In this cross-sectional study of 333 men and 498 women, we found that increased LM had a positive effect on LS and FN BMD in both men and women. On the other hand, we revealed a sex-specific association between FM and BMD as increased FM had a negative effect on FN BMD and no significant effect on LS BMD in men but a positive effect on FN and LS BMD in women. Furthermore, the subgroup analysis revealed that FM was positively associated with LS BMD only among women with low LM.

The results of our study confirm the previously reported positive impact of LM on BMD in multiple studies (7, 11–13). More importantly, our results support the recent observation from the NHANES 2011 – 2018 database that increased FM was negatively associated with total body BMD, particularly in men (0.13 lower T-score per 1 kg/m^2^ increase in FMI), which is contrary to the result from a prior meta-analysis of 44 studies demonstrating a positive association between FM and BMD (6). Notably, our findings underscore that increased FM in men may selectively affect FN BMD, rather than LS BMD, which suggests that high body fat may selectively affect cortical bone rather than trabecular bone.

Although the exact underlying mechanism of the negative impact of FM on BMD in men, but not in women, is still unclarified, it is thought to involve the effects of obesity-related hormonal changes on the skeleton (14, 15). First, obesity and increased fat mass are known to cause decreased testosterone level, an anabolic hormone that stimulates bone formation, in men due to suppression of the hypothalamic‐pituitary‐testicular axis and insulin resistance−associated reductions in sex hormone binding globulin (16, 17). Therefore, men with increased fat mass could have lower testosterone levels, which may explain the observed sex-specific association between fat mass and lower FN BMD. It is however unclear why this would selectively affect FN BMD but not LS BMD. Additionally, it should be noted that obesity is associated with increased estrogen concentrations among males and that estrogen is protective against osteoporosis in both sexes (18, 19). Data on sex hormones concentrations would have been valuable to identify the potential explanations for our observations.

Another explanation could be the difference in visceral and subcutaneous fat proportions between men and women, as previous studies have suggested that increased visceral fat may have a detrimental effect on BMD compared to subcutaneous fat due to its associated low-grade chronic systemic inflammation (increased interleukin-6 and tumor necrosis factor-α) (5). Data on body fat distribution and inflammatory markers would have been valuable to explain the difference in the results between men and women. Unfortunately, such data were not available in our study.

Other possible explanations for the inverse association between FM and BMD involve leptin, insulin resistance, vitamin D status and lifestyle factor. It has been shown that leptin-deficient and leptin-receptor deficient mice were shown to have increased bone formation, suggesting the negative effect of increased leptin in obesity on bone formation (20). Insulin resistance may also play a role in triggering bone loss, although previous studies have shown mixed results (21, 22). Furthermore, vitamin D deficiency is well-known to be associated with increased FM and obesity and therefore could mediate this association (23, 24). Finally, increased FM may represent sedentary lifestyle and lack of physical activity, which can be associated with decreased mechanical load to the skeleton and low cortical BMD (25, 26). This could particularly explain our observation of the inverse association between FMI and FN BMD in men.

Interestingly, we found that FM was positively correlated with both LS and FN BMD in women with low LM, but not in those with high LM. This suggests that LM and sex could be effect modifiers of the association between FM and BMD, which may explain the discrepancy in the results among the prior studies (6–9). The positive effect of FM on BMD could be due not only to increased mechanical load to the skeleton, but also increased estrogen produced by the adipose tissue, especially in postmenopausal women (15).

This study has certain limitations that should be acknowledged. First, data were collected retrospectively and thus factors on how DXA examinations were acquired may not have been adequately controlled, despite the established standard practice protocols in our institution. Examinations were done by several technologists, which could have some effects on the precision of the data (27), but this would, on the other hand, permit better generalizability of our finding (*e.g.* our results are generalizable regardless of the experience level or other characteristics of the technologist). In addition, the causal association cannot be concluded with certainty as this study is cross-sectional by design. Data on potential confounders and mediators, such as medical comorbidities, functional status, physical activity, vitamin D status, fat distribution, sex hormones and inflammatory markers were also not available in this study. Further prospective cohort studies with more robust adjustments are needed to confirm our observations.

## Conclusion

Our results indicate sex-specific influence of fat mass on BMD in Thais. Increased lean mass had a positive association with LS and FN BMD in both men and women. On the other hand, increased fat mass had a negative association with FN BMD and no significant association with LS BMD in men but a positive association with FN and LS BMD in women. Further prospective cohort studies are needed to draw causality of these associations.

## Data availability statement

The raw data supporting the conclusions of this article will be made available by the authors, without undue reservation.

## Ethics statement

The studies involving human participants were reviewed and approved by Khon Kaen University Human Research Ethics Committee. The patients/participants provided their written informed consent to participate in this study.

## Author contributions

All authors contributed to the article and approved the submitted version.
